# Identification of mitochondria-related key gene and association with immune cells infiltration in intervertebral disc degeneration

**DOI:** 10.3389/fgene.2023.1135767

**Published:** 2023-03-08

**Authors:** Wei Guo, Kun Mu, Wen-Shuai Li, Shun-Xing Gao, Lin-Feng Wang, Xiao-Ming Li, Jian-Yong Zhao

**Affiliations:** ^1^ Department of Orthopaedics, The Third Hospital of Hebei Medical University, Shijiazhuang, China; ^2^ Department of Orthopaedics, Hebei Province Cangzhou Hospital of Integrated Traditional Chinese Medicine-Western Medicine, Cangzhou, China; ^3^ Hebei Key Laboratory of Integrated Traditional and Western Medicine in Osteoarthrosis Research, Cangzhou, China; ^4^ Department of Breast Surgery, Hebei Province Cangzhou Hospital of Integrated Traditional Chinese Medicine-Western Medicine, Cangzhou, China

**Keywords:** MFN2, NLRP3 inflammasome, immune cells infiltration, intervertebral disc degeneration, signature genes

## Abstract

Intervertebral disc (IVD) degeneration and its inflammatory microenvironment can result in discogenic pain, which has been shown to stem from the nucleus pulposus (NP). Increasing evidence suggests that mitochondrial related genes are strictly connected to cell functionality and, importantly, it can regulate cell immune activity in response to damaged associated signals. Therefore, identification of mitochondria related genes might offer new diagnostic markers and therapeutic targets for IVD degeneration. In this study, we identified key genes involved in NP tissue immune cell infiltration during IVD degeneration by bioinformatic analysis. The key modules were screened by weighted gene co-expression network analysis (WCGNA). Characteristic genes were identified by random forest analysis. Then gene set enrichment analysis (GSEA) was used to explore the signaling pathways associated with the signature genes. Subsequently, CIBERSORT was used to classify the infiltration of immune cells. Function of the hub gene was confirmed by PCR, Western blotting and ELISA. Finally, we identified MFN2 as a crucial molecule in the process of NP cell pyroptosis and NLRP3 inflammasome activation. We speculate that the increased MFN2 expression in NP tissue along with the infiltration of CD8^+^ T cells, NK cell and neutrophils play important roles in the pathogenesis of IVD degeneration.

## Introduction

Low back pain (LBP) is a major musculoskeletal disease with adversely affects in people of all ages and socioeconomic groups (Knezevic et al., 2021). It is the most common reason for medical consultation and the leading reason of disability worldwide (Knezevic et al., 2021). LBP can be caused by a variety of reasons, but intervertebral disc (IVD) degeneration has been indicated as the most important reason (Martin et al., 2008; Livshits et al., 2011). IVD is composed of nucleus pulposus (NP) located centrally, fibrous annulus in the periphery, and cartilaginous end plates that connect cranially and caudally (Colombier et al., 2014). The healthy NP tissue serves as the hydrogel-like core of the IVD, consisting mainly of NP cells and extracellular matrix (ECM) (Lyu et al., 2019). It is generally believed that NP cells are of great important for IVD function and ECM metabolism, which first exhibits degenerative changes during IVD degeneration (Lan et al., 2021). Therefore, a further study of the molecular mechanism of NP cell loss may provide a novel therapeutic target for improving the IVD degeneration.

Mitochondria is a closed, double-membrane organelle which is found in nearly all eukaryotes. Mitochondria generate adenosine triphosphate mainly *via* oxidative phosphorylation of substances. Mitochondrial membrane potential and intimal permeability are the main characteristics of healthy mitochondria. In order to maintain oxidative phosphorylation, various carbon substrates need to be metabolized through certain pathways, and finally recycle and polymerize with tricarboxylic acid (TCA) to produce reductive equivalent. Mitochondria contain an active calcium ion transport system that activates many enzymes associated with oxidative metabolic pathways. Previous study has linked mitochondria to cell damage and a wide range of age-related diseases (Giorgi et al., 2018). Cell death induced by mitochondria is an important mechanism leading to IVD degeneration (Sun et al., 2021). It has been confirmed that disruption of mitochondrial dynamics is also closely related to mitochondrial dysfunction and oxidative stress during IVD degeneration. Xu et al. (2019) found that the accumulation of progerin in human NP tissues was correlated with IVD degeneration progression, and further studies confirmed that progerin stimulation could shift mitochondrial dynamics to fission events by reducing the levels of mitochondrial fusion factors Opa1 and increasing the levels of mitochondrial fission factor Drp1 (Song et al., 2021). NP cell function loss occurs in the IVD degeneration progression stage due to cell senescence, death, inflammatory responses, and imbalances in anabolic and catabolic metabolism, which are also closely associated with mitochondrial damage (Wang et al., 2020a; Huang et al., 2020). Most studies on mitochondria-related genes focus on their regulation of mitochondrial function (Prasun, 2020; Sun et al., 2021). However, increasing evidence suggests that mitochondrial related genes are strictly connected to cell functionality and, importantly, it can regulate cell immune activity in response to damaged associated signals (Zhang et al., 2021; Deng et al., 2022). IVD was considered as an immunologically privileged organ that isolated NP tissue from the host immune system due to their unique structure. When the blood-NP barrier is breached, NP tissue triggers an immune response. This process plays an important role in IVD degeneration and result in a variety of subsequent pathological processes (Bridgen et al., 2017). Therefore, the relationship of mitochondrial related genes with NP cell inflammatory response in the process of IVD degeneration has become a research hotspot.

In this study, bioinformatics analysis based on transcriptome microarrays was used to identify mitochondria-associated genes in patients with IVD degeneration, providing new insights into the diagnosis and treatment of the disease. Moreover, we investigated the effects of Mitofusin-2 (MFN2) overexpression on human NP cells pyroptosis and NLRP3 inflammasome activation.

## Materials and methods

### Ethics statement

This study was approved by the ethics committees of Hebei Province Cangzhou Hospital of Integrated Traditional Chinese Medicine-Western Medicine (2020022). Human NP tissue samples were obtained from patients undergoing surgery at Hebei Province Cangzhou Hospital of Integrated Traditional Chinese Medicine-Western Medicine. Written informed consent was obtained from all patients for the use of their tissue specimens for research purpose.

### Clinical specimens

Human lumbar degenerative NP specimens were obtained from 10 patients with IVD degeneration undergoing discectomy. The control samples were taken from 10 age- and sex-matched patients with fresh traumatic vertebral fracture undergoing decompressive surgery because of neurological deficits.

### Data sources

In this study, two data sets were downloaded from the Gene Expression Omnibus (GEO) (http://www.ncbi.nlm.nih.gov/geo/), namely, GSE56081 [platform: GPL15314 Arraystar Human LncRNA microarray V2.0 (Agilent-033010 probe name version) and GSE165722 (GPL24676 Illumina NovaSeq) 6,000 (*Homo sapiens*)]. GSE56081 contained two groups of mRNA expression profiles, containing 5 NP tissue from IVD degeneration patients and five from controls. GSE165722 contained eight samples of Single-cell transcriptome profiling from NP tissue. A total of 1,136 mitochondria-related genes (Mito-Genes) were downloaded from the Mitocarta3.0 database (Rath et al., 2021).

### Weighted gene coexpression network analysis (WGCNA) was used to identify IVD degeneration genes

For this study, a R package named “WGCNA” was used to construct the co-expressed clusters by analysis the gene expression level of GSE56081 (Langfelder and Horvath, 2008). Prior to the network construction, the raw data was converted into a recognizable format using R package “affy,” then the missing values were estimated using k nearest neighbor based approach, and then the median method was used for normalization (Troyanskaya et al., 2001). The pickSoftThreshold function of WGCNA package is used to calculate the soft threshold power and adjacency relationship. Then, the adjacency matrix is converted into topological overlap matrix, and the corresponding dissimilarity is calculated to carry out hierarchical clustering analysis. The dynamic tree cutting method with a minimum module size of 20 were used to identify the co-expressed gene modules. The module associated with IVD degeneration was identified by correlation analysis. IVD degeneration genes were acquired by module membership within the modules.

### Identification of IVD degeneration-mitochondria related genes

The IVD degeneration-mitochondria related genes (IVDD-Mito_Genes) were obtained by intersecting the IVD degeneration genes and Mito-Genes. We then used R package clusterProfiler to analyze the GO and KEGG signaling pathways of IVDD-Mito_Genes and output the top 10 GO and 15 KEGG signaling pathways.

### Signature genes identification

Subsequently, we exerted machine learning algorithms called random forest to screen IVDD-Mito_Genes. We used R packet “randomforest” to classify IVDD-Mito_Genes. The random forest model calculated the average error rate of IVDD-Mito_Genes to determine the optimal number of variables (Xie et al., 2022; Yu et al., 2022). We then calculate the error rate for each tree and determine the optimal number of trees based on the lowest error rate. After the above parameters are determined, the random forest tree model is established. Finally, the characteristic importance score of each IVDD-Mito_Genes was performed, and the top10 genes were selected. We used the area under the curve (AUC) of the receiver operating characteristic curve (ROC) to evaluate the diagnostic efficiency of these signature genes. An AUC greater than 0.8 indicates a good diagnostic effect.

### Gene set enrichment analysis

We grouped the IVD degeneration cohort according to the median expression of signature genes, and performed gene set enrichment analysis (GSEA) according to different subgroups to determine the relationship between signature genes and signaling pathways (Subramanian et al., 2005).

### Immune cell infiltration analysis

We used the principle of linear support vector regression to deconvolution the expression matrix of 22 human immune cell subtypes by CIBERSORT method to investigate the differences of immune cell between IVD degeneration patients and normal subjects. Subsequently, immune cells with significant differences in infiltration between patients with IVD degeneration and normal subjects were screened, and spearman correlation analysis was used to identify their correlation with signature genes.

### Signal cell RNA-seq data analysis

Raw RNA-seq data (GSE165722) were processed according to bioinformatics analysis principles. Cells with a unique feature count of <200 and a mitochondrial count of >20% in the sample were filtered out. t-distributed stochastic neighbor embedding (t-SNE) analysis, K-mean clustering and hierarchical clustering methods were used to analyze the data. The following R packages were used: limma, Seurat, dplyr, magrittr, celldex, SingleR, monocle.

### Cell communication analysis

Cell communication is determined by assessing the expression of ligand and receptor pairs within a cell population (CellChat R package). Interactions between different cell types were examined, and gene expression 0.2 was set as an effective cut-off point.

### Human NP cell culture

The NP tissue samples were separated and cut into 1 m pieces, and the nucleus pulposum tissue was digested with 0.25% pronase and 0.2% collagenase type II at 37°C. The digested suspension was filtered through a 70 μm pore mesh and cultured in DMEM medium containing 10% fetal bovine serum and 1% penicillin-streptomycin at a CO_2_ concentration of 5% and a temperature of 37°C.

### Western blotting

Cells were lysed with a buffer containing a protease inhibitor, 0.25 M Tris-HCl, 20% glycerol, 4% sodium dodecyl sulfate (SDS), and 10% mercaptoethanol (pH 6.8). The same amount of total protein (10 μg) was separated by 10%–12% SDS-polyacrylamide gel and electrotransferred to polyvinylidene fluoride membrane. Then, 5% non-fat milk in Tris-buffered saline containing 0.1% Tween-20 (TBST) was employed for blocking at ambient (1 h), followed by incubation with primary antibodies in TBST containing 5% non-fat milk overnight at 4°C. Secondary antibody was added at room temperature for 1 h and Western blotting was performed using an enhanced chemiluminescence system.

### ELISA

The contents of human IL-1β in the cell culture supernatant were detected according to the manufacturer’s instructions.

### Quantitative reverse-transcription PCR (RT-qPCR)

After chloroform extraction and precipitation, the DNA in the sample was removed by DNase I treatment, followed by reverse transcription of purified RNA using RevertAid reverse transcriptase. The reverse transcription primers containing CMV promoter sequences were designed to target specific genes. RT-qPCR was performed with AriaMx Real-time PCR system and QuantStudio Real-time PCR system. Supplementary Table S3 showed the primers used in this study.

### Cell transfection

Lipofectamine 3,000 (Invitrogen) was used to transfect the plasmids or siRNA respectively with third-generation NP cells as recommended by the manufacturer and described previously (Guo et al., 2020). Cells were collected 48 h after transfection.

### Statistical analysis

All experiments were repeated three times or more. Continuous data are mean ± standard deviation (SD). Bioinformatic analyses in this study was performed by R software (version 4.1.2). Prism 7.0 (GraphPad Software, United States) and SPSS 22.0 (SPSS Inc., United States) were used for statistical analyses. *p < 0.05, **p < 0.01, and ***p < 0.001 were significance levels.

## Results

### Identification of IVD degeneration genes by WGCNA analysis

**FIGURE 1:**
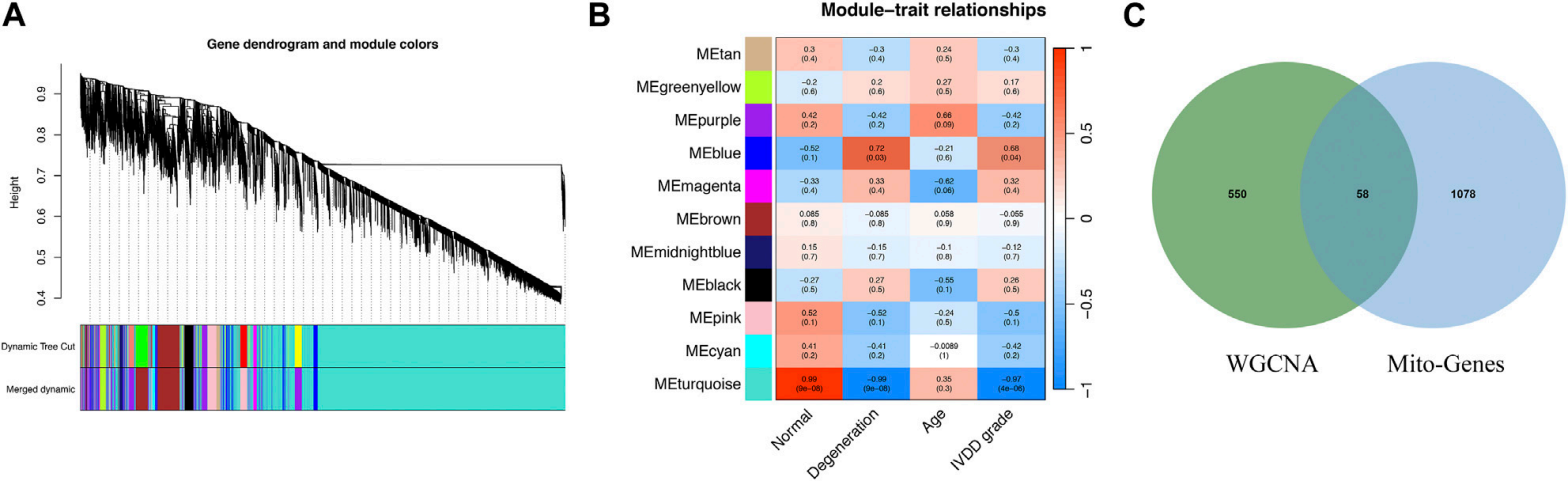
The gene modules significantly associated with IVD degeneration were identified by WGCNA. **(A)** WGCNA cluster dendrogram. **(B)** WGCNA cluster modules. **(C)** Venn diagram shows the interaction between mitochondria-associated genes and genes associated with IVD degradation.

We use dynamic shear tree algorithm to segment the modules, and set the minimum size of the modules as 20 to build a scale-free co-representation network. Figure 1A shows the results of the cluster tree. Finally, the data were clustered into 19 modules (Figure 1B). Then the correlation between each module and IVD degradation is calculated. Supplementary Table S1 shows the genes in each module. Correlation analysis showed that blue module was significantly associated with IVD degradation. Therefore, genes in blue module was considered as IVD degradation genes. The crossover between the IVD degeneration genes and mitochondrial related genes was shown in Figure 1C (Supplementary Table S2).

### Function enrichment analysis

**FIGURE 2:**
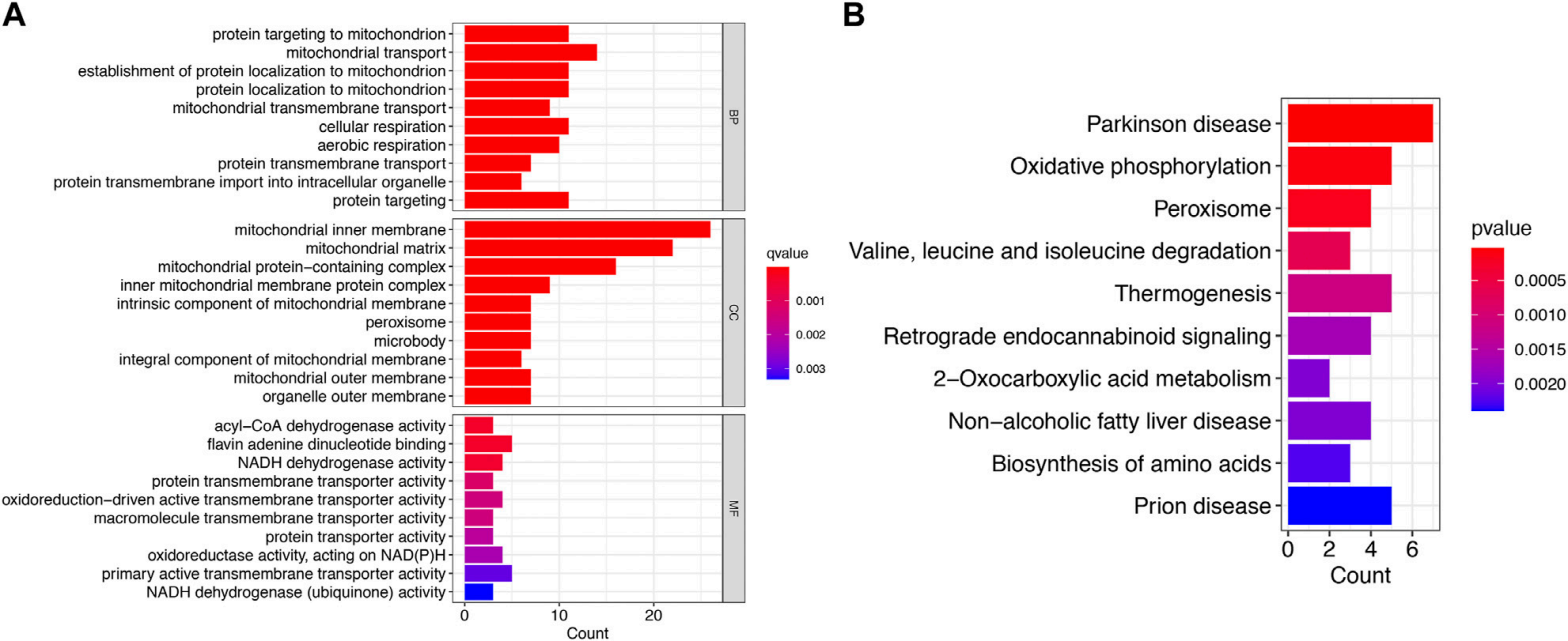
Functional enrichment analysis of IVD degeneration-Mito_Genes. **(A)** Top 10 BP, MF, CC of GO functional enrichment analysis. **(B)** The KEGG analysis of degeneration-Mito_Genes.

By GO and KEGG enrichment analysis of 58 IVD degeneration-Mito_genes, 81 biological processes (BP), 50 cellular components (CCs), 32 molecular functions (mf) and 20 KEGG signaling pathways were identified. The enrichment analysis results of the top 10 GO and 10 KEGG signaling pathways are shown in Figures 2A, B, respectively. The results showed that these genes are enriched in biological processes such as protein targeting to mitochondrial, mitochondrial transport, mitochondrial protein localization, protein transmembrane transport, and KEGG signaling pathways such as those in Parkinson’s disease, and oxidative phosphorylation.

### Identification of signature genes by random forest algorithms

**FIGURE 3:**
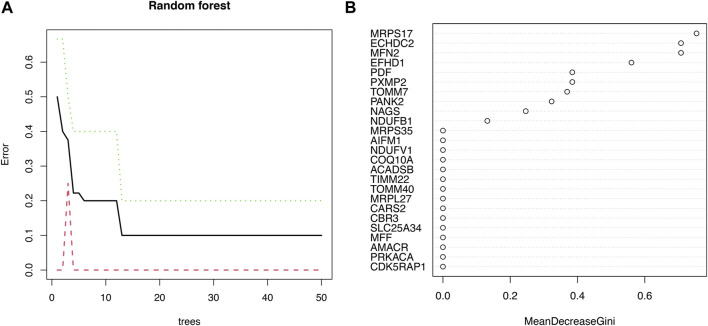
The machine algorithms for signature genes. **(A)** Confidence intervals for error rates of random forest models. **(B)** The relative importance of genes in random forest models.

Eight signature genes with relative importance greater than 0.3 were identified by random forest analysis (Figures 3A, B), containing MRPS17, ECHDC2, MFN2, EFHD1, PDF, PXMP2, TOMM7, PANK2.

### Diagnostic efficacy of signature genes in predicting IVD degeneration

**FIGURE 4:**
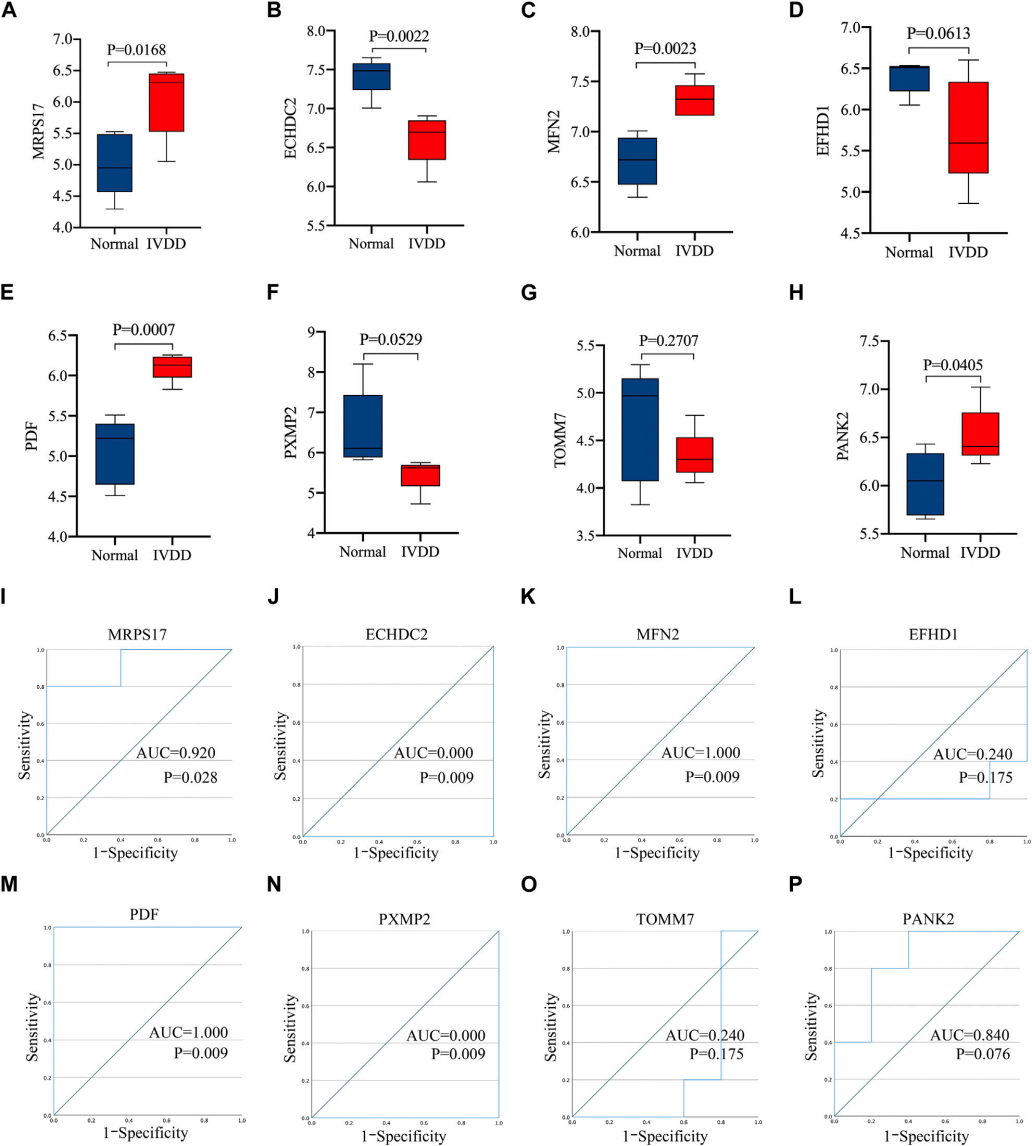
The performance of the signature genes. **(A–H)** Signature gene expression levels in the nucleus pulposus tissue of IVD degeneration patient and healthy subjects. **(I–P)** The diagnostic performance of signature genes was evaluated by ROC.

Figures 4A–H showed that four of the signature genes screened through random forest were more highly expressed in patients with IVD degeneration than normal people, including MRPS17, MFN2, PDF and PANK2, suggesting that these genes may play a key role in IVD degeneration. Figures 4I–P shows the area under curve (AUC) of these signature genes. The results showed that three of the signature genes (MRPS17, MFN2, PDF) had significant diagnostic effect in predicting the degeneration of IVD. These results were further confirmed in 10 patients and 10 controls human NP samples using RT-qPCR analysis and Western blotting, MRPS17, MFN2, PDF were significantly increased in IVD degeneration NP tissues, MFN2 showed significant diagnostic effect in predicting IVD degeneration (Supplementary Figure S1).

### Gene set enrichment analysis

**FIGURE 5:**
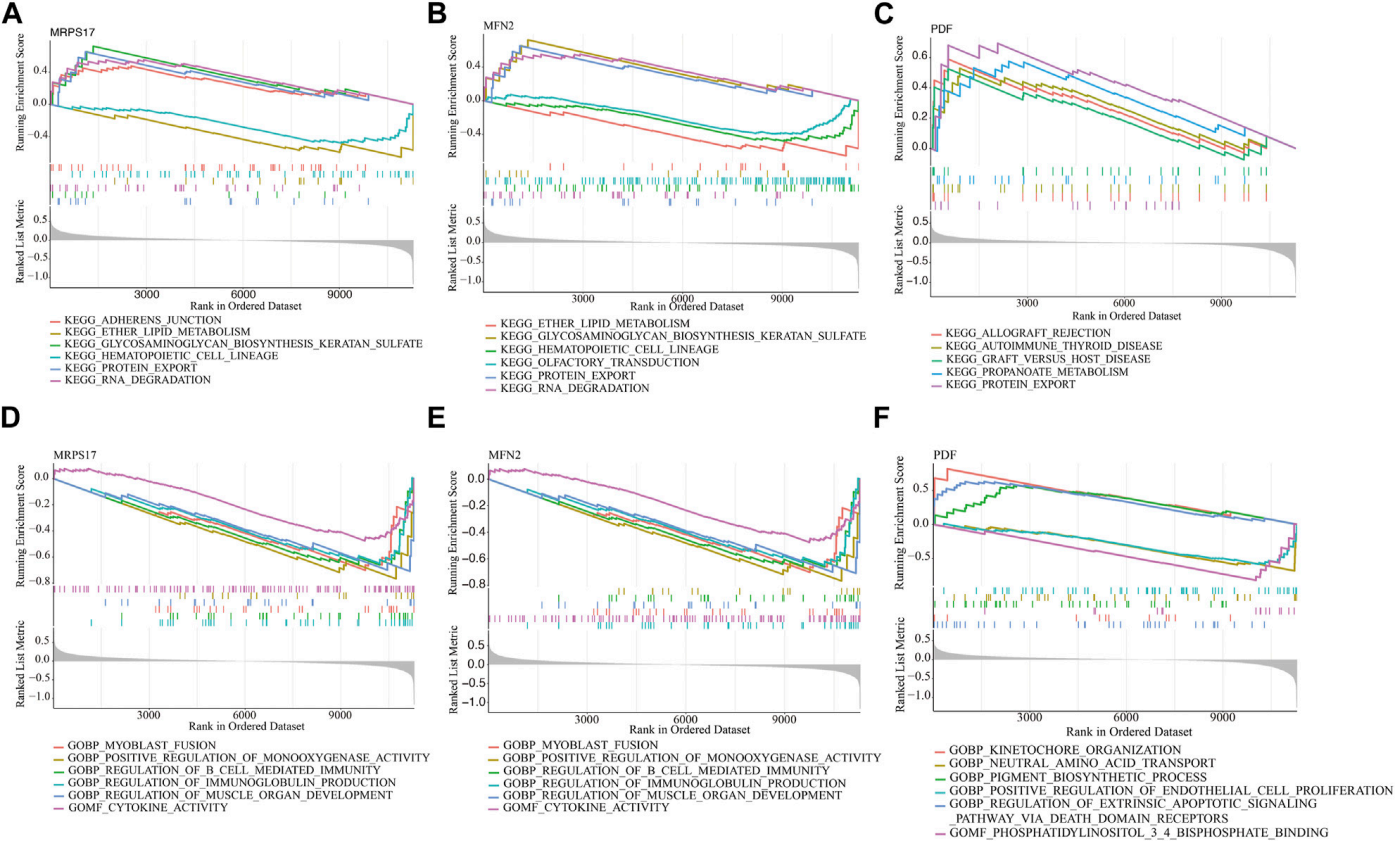
The GSEA of the signature genes in IVD degeneration. **(A–C)** KEGG enrichment results of signature genes (MRPS17, MFN2, PDF). **(D–F)** GO enrichment results of signature genes (MRPS17, MFN2, PDF).

We analysis signaling pathways and GO associated with signature genes which had diagnostic efficiency by GSEA analysis. Figures 5A–C demonstrated the top six signal pathways. The results showed that MRPS17 was markedly associated with adherens junction, ether lipid metabolism, biosynthesis keratan sulfate, hematopoietic cell lineage, protein export, RNA degradation (Figure 5A). The expression of MFN2 significantly correlated with ether lipid metabolism, biosynthesis keratan sulfate, hematopoietic cell lineage, olfactory transduction, protein export and RNA degradation (Figure 5B). The expression of PDF significantly correlated with allograft rejection, autoimmune thyroid disease, graft versus host disease, propanoate metabolism and protein export (Figure 5C). The top six GO were demonstrated in Figures 5D–F. The results showed that MRPS17 was significantly correlated with myoblast fusion, positive regulation of monooxygenase activity, regulation of B cell mediated immunity, regulation of immunoglobulin production, regulation of muscle organ development and cytokine activity (Figure 5D). The expression of MFN2 significantly correlated with myoblast fusion, positive regulation of monooxygenase activity, regulation of B cell mediated immunity, regulation of immunoglobulin production, regulation of muscle organ development and cytokine activity (Figure 5E). The expression of PDF significantly correlated with kinetochore organization, neutral amino acid transport, pigment biosynthetic process, positive regulation of endothelial cell proliferation, regulation of extrinsic apoptotic signaling pathway *via* death domain receptors (Figure 5F).

### Immune cell infiltration

**FIGURE 6:**
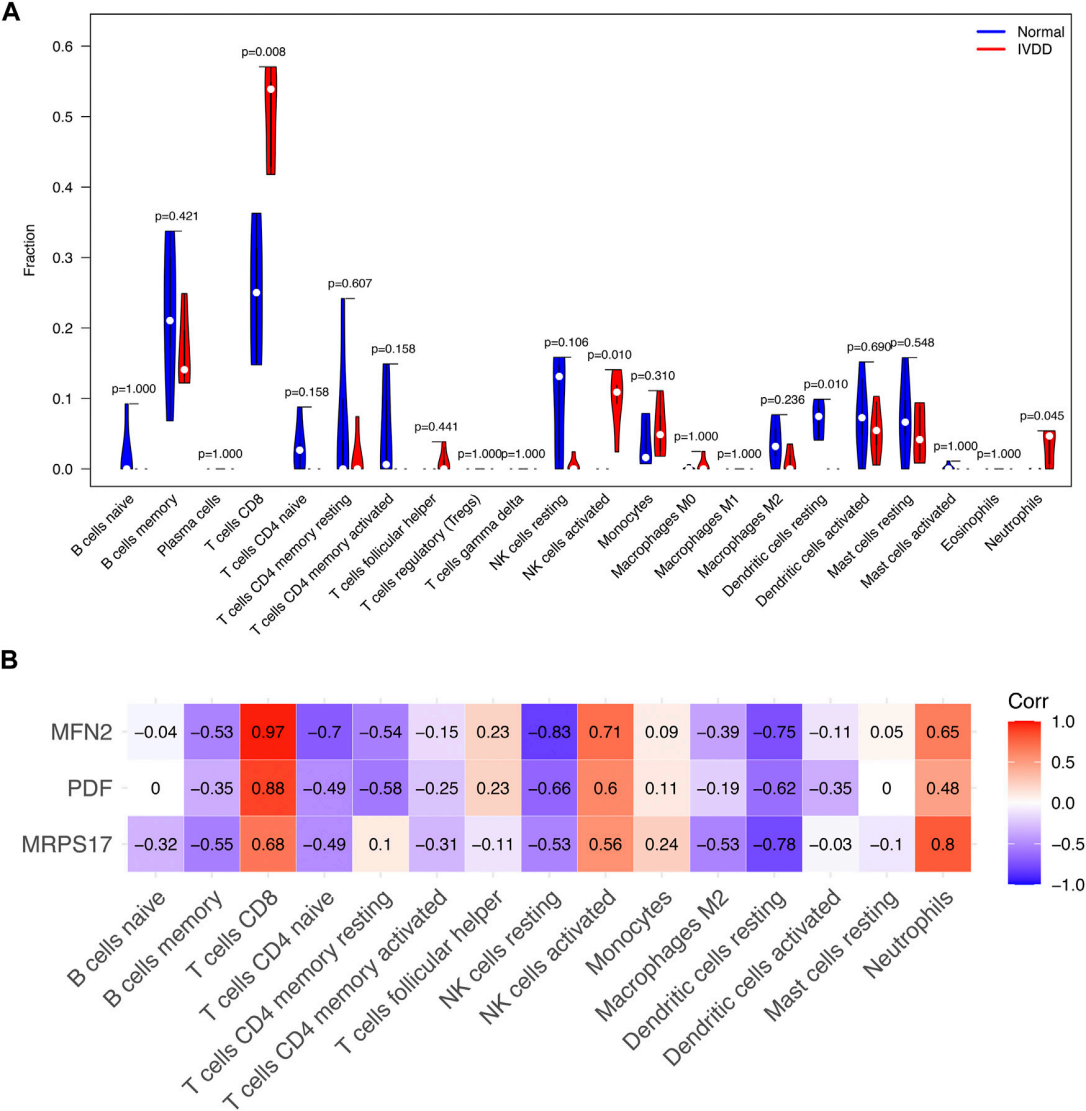
Signature genes related to immune cell infiltration. **(A)** The infiltration of immune cells in the NP tissues of the IVD degeneration and healthy subjects. **(B)** The relationship between signature genes and immune cell infiltration.

Compared with normal subjects, IVD degeneration patients have higher CD8^+^ T cells, NK cell activated, neutrophils infiltration and lower dendritic cell resting (Figure 6A). MFN2 was associated with the infiltration of CD8^+^ T cells, NK cell activated and neutrophils positively. PDF was associated with the infiltration of CD8^+^ T cells positively. MRPS17 was positively associated with the infiltration of CD8^+^ T cells and neutrophils (Figure 6B). Notably, MFN2 was positively correlated with all the IVD degeneration related immune cell infiltration. These results demonstrated that MFN2 might be a potential immune-related target, and further study of MFN2 might provide a better understanding of immune cell infiltration in IVD degeneration.

### Single-cell transcriptome profiling of the IVD tissue

**FIGURE 7:**
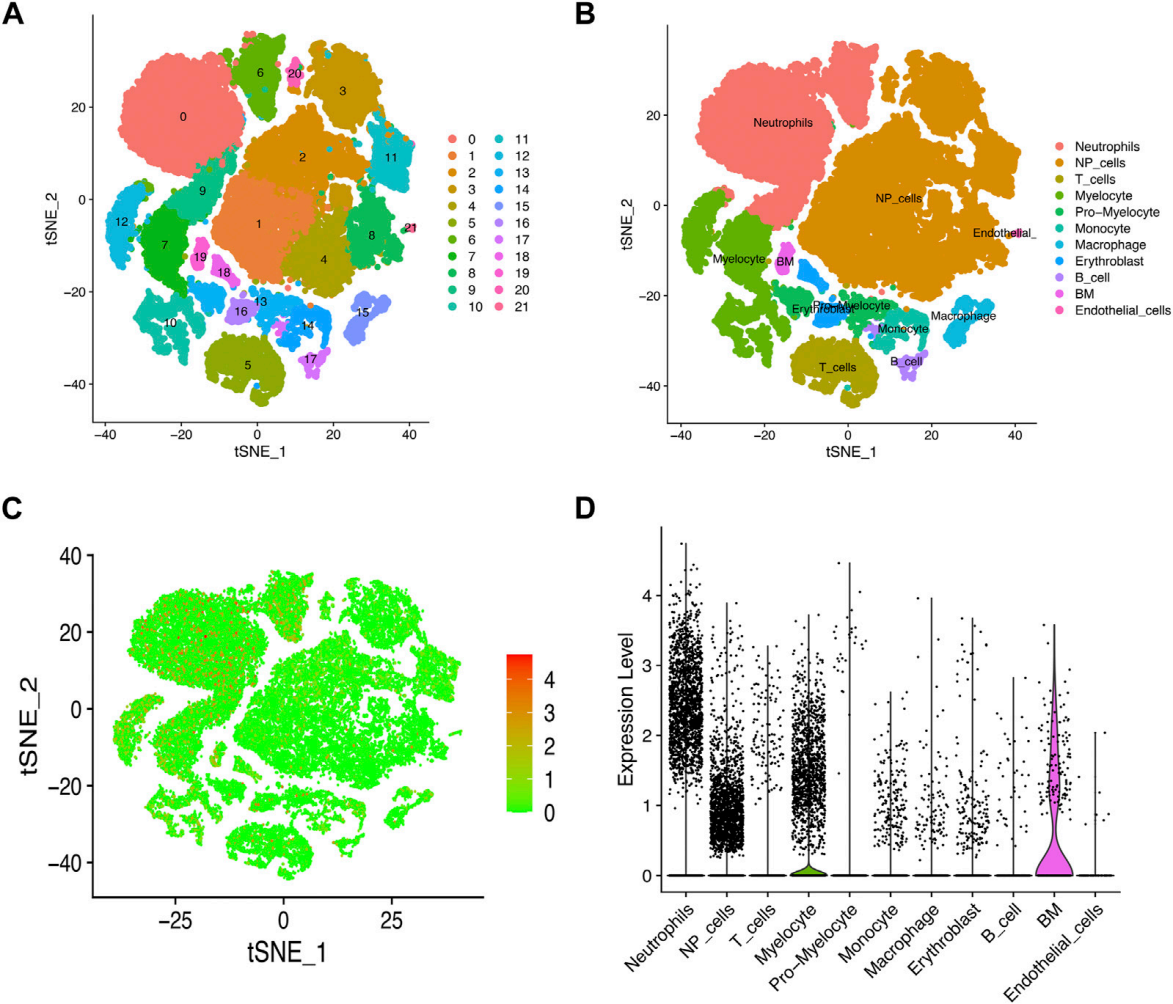
Single cell sequencing data analysis and identification of different cell types. **(A, B)** t-SNE projections and cell type annotation of IVD tissue. **(C, D)** t-SNE and violin diagrams demonstrated MFN2 expression pattern at the single-cell level.

We used scRNA-seq data (GSE165722) to analyze and identify different cell types in NP tissues during IVD degeneration, and visualized the results with dimensionality reduction algorithm (t-SNE). We identified 21 cluster and 11 major distinct cell types which were determined using singleR and cell markers (Figures 7A, B). Consistent with above results, we found a lot of immune cells in the tSNE map which may suggest the immune cell infiltration during the progress of IVD degeneration. Subsequently, we examined MFN2 expression in these cell types. As expected, MFN2 was expressed in NP cells and most immune cells (Figures 7C, D).

**FIGURE 8:**
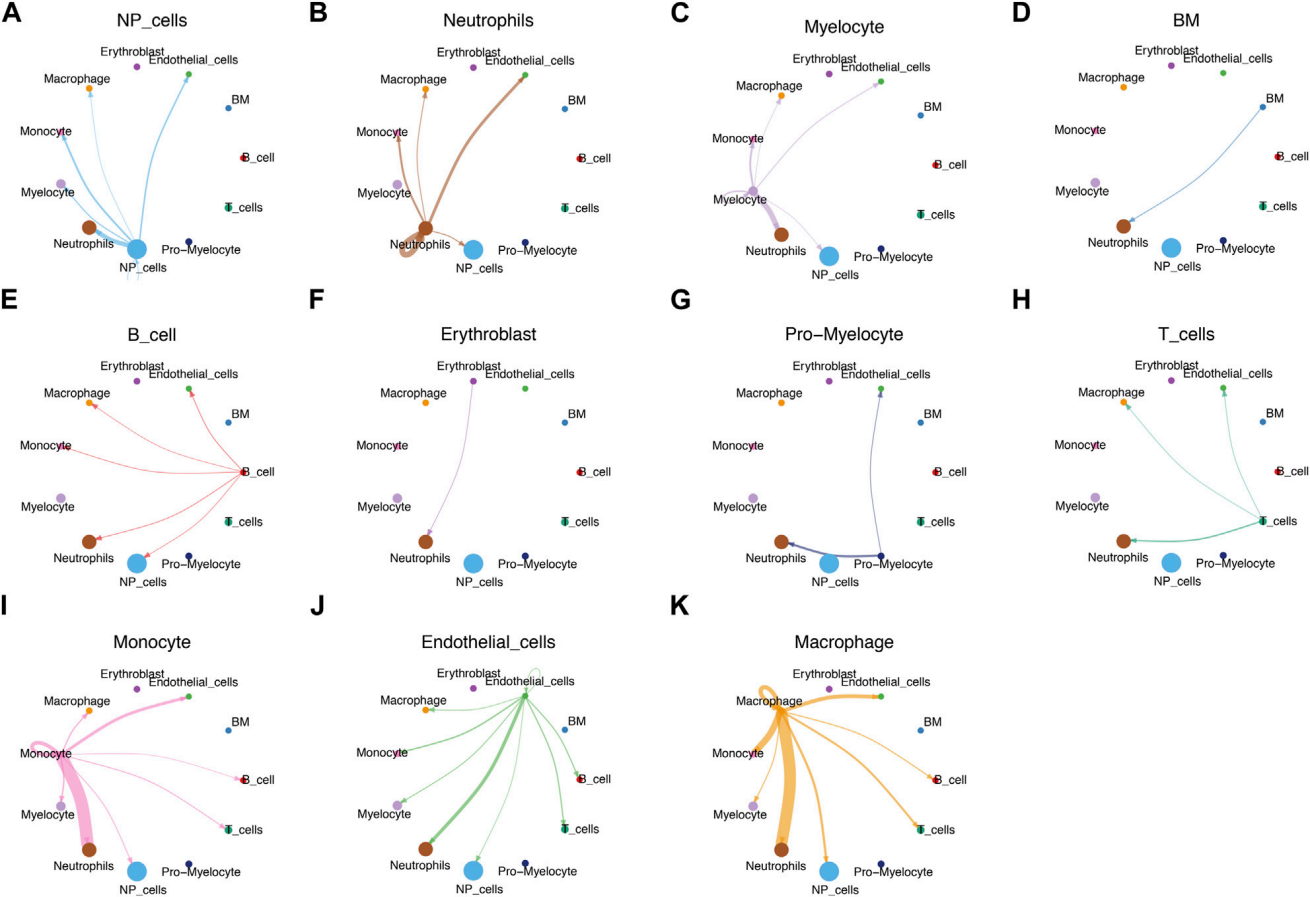
CellChat analysis. **(A–K)** Circle plots demonstrated the interactions in the cell-cell communication network between IVD degeneration and healthy subjects, respectively.

We used CellChat analysis to identified potential interactions between different types of immune cells and NP cells. The results showed that NP cells interact with neutrophils, myelocyte, monocyte, macrophage, endothelial cells closely (Figures 8A–K).

### Mfn2 overexpression activated NLRP3 inflammasome

**FIGURE 9:**
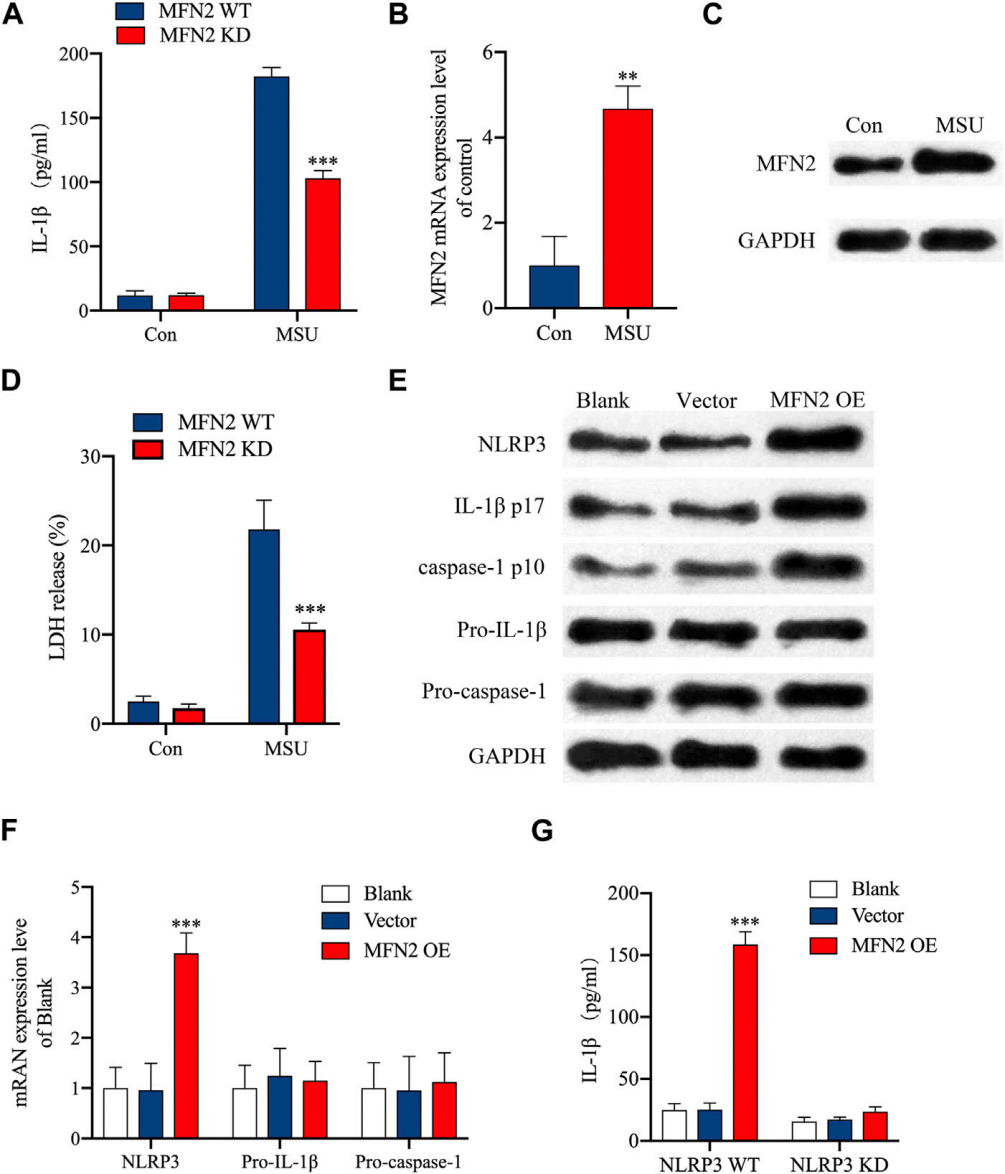
Overexpression of MFN2 activated NLRP3 inflammasome in NP cells. **(A)** NP cells were transfected as indicated. IL-1β released was assessed by ELISA. **(B, C)** NP cells were stimulated with MSU. MFN2 mRNA expression was assessed by RT-qPCR and Western blot. **(D)** Pyroptosis was determined by LDH assay. **(E)** The Western blot assay for NLRP3, IL-1β cleavage and caspase-1 activation. **(F)** NP cells were transfected as indicated. NLRP3, pro- IL-1β and pro-caspase-1 mRNA were assessed by RT-qPCR. **(G)** NP cells were transfected as indicated. IL-1β released was assessed by ELISA.

IL-1β secretion following exposure to MSU (a prototypical danger signals) was significantly decreased in MFN2 knockdown NP cells as compared to wild-type NP cells (Figure 9A). As shown in Figures 9B, C, MSU increased MFN2 mRNA and protein expression level in comparison with control group. Pyrosis is a programmed inflammatory cell death that occurs when inflammasome is activated. In this study, we found that the absence of MFN2 markedly attenuated the release of LDH from NP cells, suggesting that MFN2 had a regulatory effect on pyroptosis (Figure 9D). To elucidate the effect of MFN2 in the activation of NLRP3 inflammasome, we examined IL-1β cleavage and caspase-1 activation in MFN2 overexpressed NP cells. The results showed that the activation of Caspase-1 and the cleavage of IL-1β were significantly increased in MFN2 overexpressed NP cells (Figure 9E). Notably, MFN2 overexpression significantly increased NLRP3 protein and mRNA expression level, but had no effect on pro-IL-1β and pro-caspase-1 expression (Figures 9E, F). And NLRP3 knockdown markedly inhibited IL-1β secretion in MFN2 overexpression NP cells (Figure 9G). These results indicated that MFN2 can promote the expression of NLPR3 and activate the NLRP3 inflammasome, resulting in the pyroptosis of NP cells and the release of inflammatory cytokines.

## Discussion

MFN2 is a multifunctional protein involved in a variety of physiological and pathological processes, including mitochondrial fusion transport, cell metabolism, pyroptssis, and autophagy (Yu et al., 2018). Dysregulation of MFN2 can lead to neurodegeneration, sarcopenia, metabolic disease, heart disease and many other diseases (Sebastian et al., 2016; Kim et al., 2017; Chandhok et al., 2018; Rocha et al., 2018). Increasing evidence have demonstrated that immune cell infiltration acts as an important role in IVD degeneration (Wang et al., 2021). This study is the first time to reveal the relationship between MFN2 and immune cell infiltration during intervertebral disc degeneration.

Previous studies have shown that MFN2 can be phosphorylated by Jun N-terminal kinase under cellular stress, leading to MFN2 degradation through the ubiquitin-proteasome system (Leboucher et al., 2012). The ubiquitination of MFN2 promotes mitochondrial degradation and prevents fusion of damaged mitochondria (Tanaka et al., 2010). In this study, our results indicated that MFN2 has the ability to regulate immune response in addition to the function of regulating mitochondrial fusion. Previously research demonstrated that IVD degeneration is characterized by infiltration of CD68^+^ macrophages, T cells (CD4^+^, CD8^+^), and neutrophils (Wang et al., 2021). In combination with scRNA-seq, cell heterogeneity can be identified for in-depth study of biological structure and function. The scRNA-seq technique can generate the expression profile of individual cells for the analysis of heterogeneous cell populations and the identification of cell types. Understanding the phenotype of immune cells in the IVD microenvironment is critical to understanding the mechanisms of IVD degeneration progression. Immune infiltrating cells in the IVD degeneration have been shown to be very important in the IVD degeneration (Risbud and Shapiro, 2014; Wang et al., 2021). In this research, we found significantly increased infiltration of CD8^+^ T cells and neutrophils in degenerative NP tissues compared to controls. We also found a strong positive correlation between the MFN2 and the level of immune infiltration of three types of invasive immune cells (including CD8^+^ T cells, NK cell and neutrophils). This implied a strong association of MFN2 with immune cells in the IVD degeneration, which was subsequently validated using scRNA-seq datasets. CellChat can quantitatively infer and analyze intercellular communication networks from scRNA-seq data. Our results showed the number of interactions between NP cells and immune cells in the IVD degeneration including neutrophils, myelocyte, monocyte, macrophage. IVD is considered as an immunologically privileged organ because their unique structure insulates the NP tissue from the host immune system (Sun et al., 2020). When NP cells pyroptosis, NP releases inflammatory factors such as IL-1β, which induces immune cell infiltration and triggers an immune response. This process act as an important role in IVD degeneration and result in multiple pathological processes that eventually lead to fibrotic of NP tissue (Wang et al., 2020b). At the same time, immune cells recruited into the IVD microenvironment will release more inflammatory mediators, further damaging the IVD microenvironment and causing nucleus pulposus cell death (Silva et al., 2019).

NLRP3 is a member of the pattern recognition receptors that mediate the activation of the innate immune system. The NLRP3 inflammasome is important for the immune defense system. NLRP3 initiates inflammasome assembly, promotes recruitment of inflammasome complex by caspase-1 and activates caspase-1. Activated caspase-1 cleave the IL-1β precursor protein and converts it into a biologically active mature form, which promotes an immune response. Previous studies have shown that MFN2 acts as a major regulator of the immune response by binding to NLRP3 and promoting IL-1β secretion after infection with the virus (Ichinohe et al., 2013; Tur et al., 2020). In our research, we observed that MFN2 was highly expressed during IVD degeneration. MFN2 overexpression associated with the infiltration of CD8^+^ T cells, NK cell activated and neutrophils. MFN2 overexpression also activate NLRP3 inflammasome which results in IL-1β release and NP cell pyroptosis. This may be due to the overexpression of MFN2 in NP cells inducing the expression of NLRP3 and the activation of inflammasome. Combining the results of this study with the conclusions of previous studies, we hypothesized that a special type of immune microenvironment is generated during the process of IVD degeneration by release of IL-1β from NP cells that recruit CD8^+^ T cells, NK cell activated and neutrophils. This immune microenvironment, in turn, further promotes NP cell death and IVD pathological changes.

This study also has some limitations. First of all, the data in this study are mainly from public databases and involve a small number of samples, which may lead to certain bias. However, the reliability of our analysis was confirmed by the results of *in vitro* experiments. Second, this study still needs a larger clinical samples to further validate the results. Third, the specific mechanism of MFN2 cause NLRP3 induction and increase inflammasome activation remains to be explored.

Our research shows that immune cell infiltration, including T cells, NK cell activated and neutrophils, is participated in the pathological process of IVD degeneration. Via microarray data analysis, single-cell sequencing data analysis and *in vitro* experiments, we identified MFN2 as a signature gene, which present good diagnostic value and may serve as a molecular target for treatment of IVD degeneration.

## Data Availability

The original contributions presented in the study are included in the article/Supplementary Material, further inquiries can be directed to the corresponding authors.

## Supplementary material

SUPPLEMENTARY FIGURE S1
**(A)** The mRNA expression level of signature genes in NP tissues was measured in 10 patients and 10 controls using qRT-PCR (**P < 0.01; ***P < 0.001). **(B–I)** The diagnostic performance of signature genes was evaluated by ROC. **(J–Q)** The protein expression level of signature genes in NP tissue were measured by Western blotting.

SUPPLEMENTARY FIGURE S2 The efficiency of MFN2 knock down in NP cells.
