# Supplementary figures and images for: Identification of mitochondria-related key gene and association with immune cells infiltration in intervertebral disc degeneration
Source: Front Genet. 2023 Mar 8;14:1135767. doi: 10.3389/fgene.2023.1135767 (PMC10030706; doi:10.3389/fgene.2023.1135767)

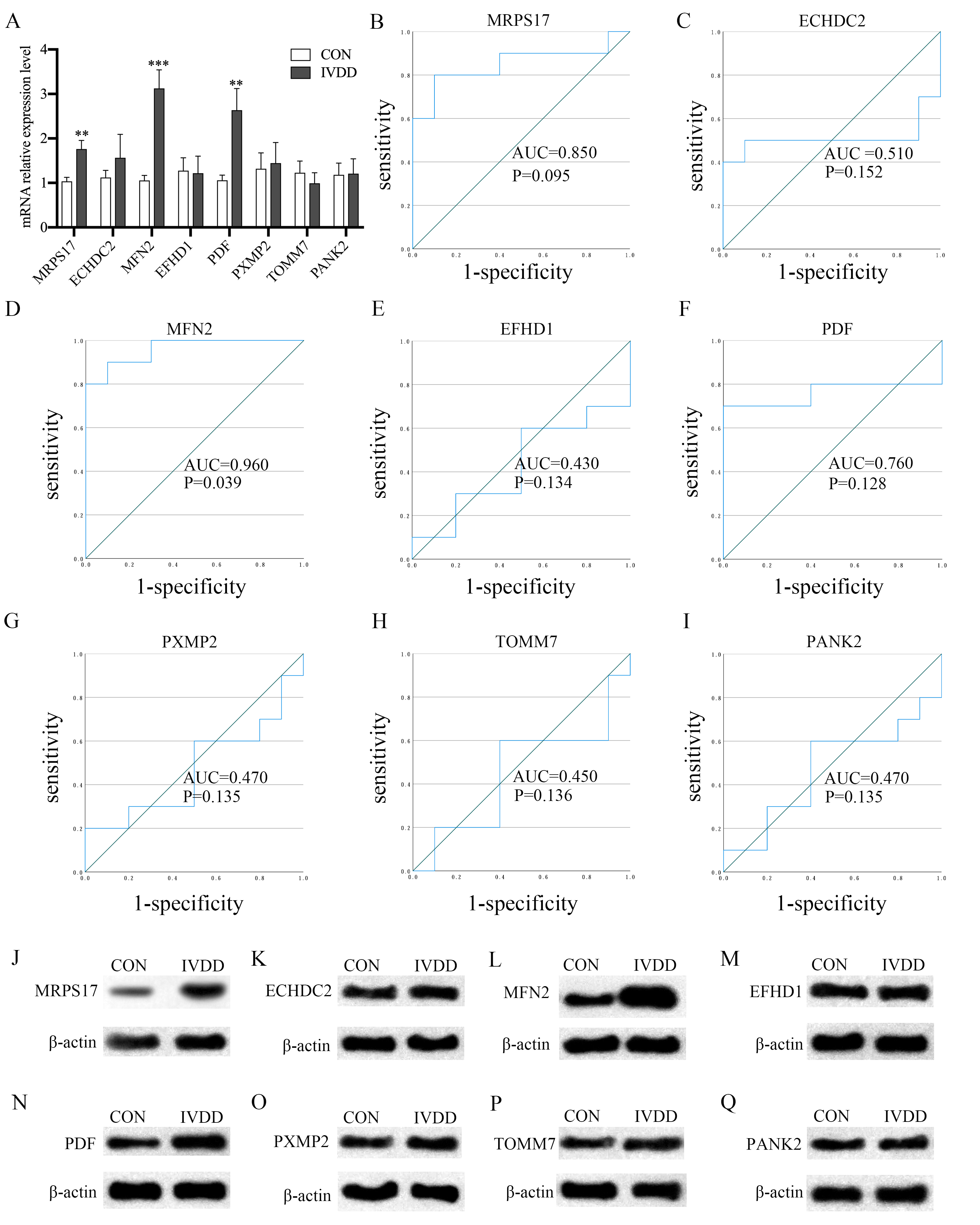

Supplement: Supplementary file 2 [file Image1.TIF]

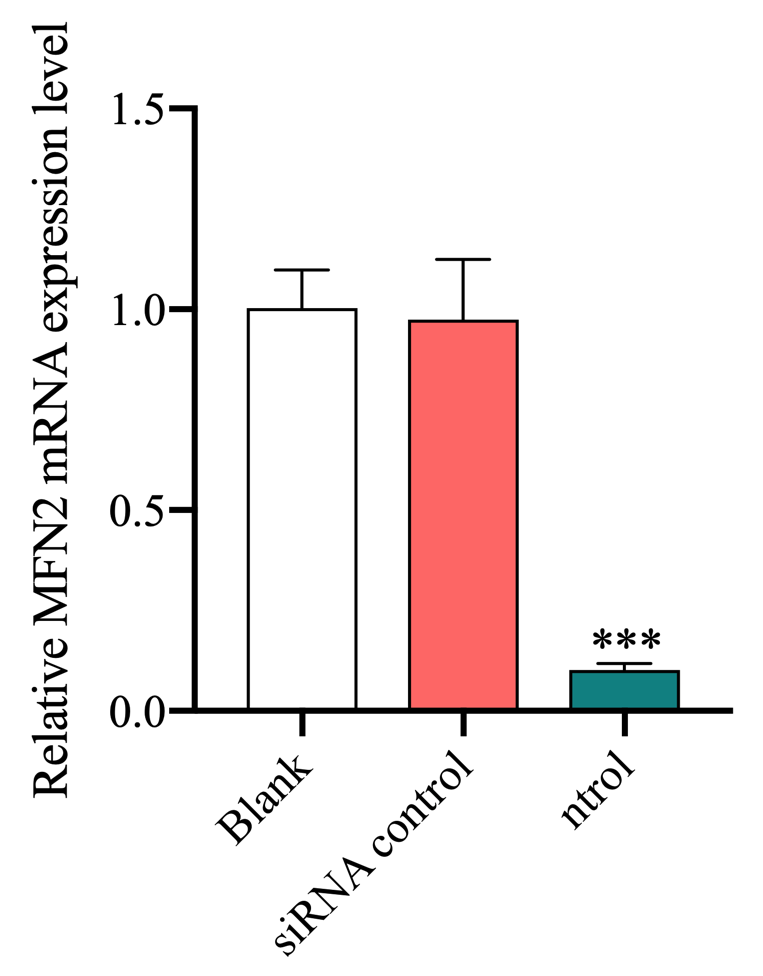

Supplement: Supplementary file 6 [file Image2.TIFF]
