# Supplementary material for: Identification of mitochondria-related key gene and association with immune cells infiltration in intervertebral disc degeneration
Source: Front Genet. 2023 Mar 8;14:1135767. doi: 10.3389/fgene.2023.1135767 (PMC10030706; doi:10.3389/fgene.2023.1135767)
Supplement: Supplementary file 3 [file Table3.DOCX]

**Table S3. Primer sequence for RT-qPCR**

| Gene | Forward Primer (5′-3′) | Reverse Primer (5′-3′) |
| --- | --- | --- |
| MFN2 | CTCTCGATGCAACTCTATCGTC | TCCTGTACGTGTCTTCAAGGAA |
| NLRP3 | GATCTTCGCTGCGATCAACAG | CGTGCATTATCTGAACCCCAC |
| Pro-IL-1β | ATGATGGCTTATTACAGTGGCAA | GTCGGAGATTCGTAGCTGGA |
| Pro-caspase-1 | TTTCCGCAAGGTTCGATTTTCA | GGCATCTGCGCTCTACCATC |
| GAPDH | ACAACTTTGGTATCGTGGAAGG | GCCATCACGCCACAGTTTC |
