# Supplementary material for: Identification of mitochondria-related key gene and association with immune cells infiltration in intervertebral disc degeneration
Source: Front Genet. 2023 Mar 8;14:1135767. doi: 10.3389/fgene.2023.1135767 (PMC10030706; doi:10.3389/fgene.2023.1135767)
Supplement: Supplementary file 5 [file Table4.DOCX]

Table S4. Demographic information of GSE56081

| Sample | Age | Gender | Pfirrmann grade |
| --- | --- | --- | --- |
| GSM1354764 | 33 | Male | 1 |
| GSM1354765 | 35 | Male | 1 |
| GSM1354766 | 41 | Male | 1 |
| GSM1354767 | 43 | Female | 1 |
| GSM1354768 | 52 | Male | 1 |
| GSM1354769 | 32 | Female | 5 |
| GSM1354770 | 38 | Male | 5 |
| GSM1354771 | 42 | Male | 4 |
| GSM1354772 | 45 | Male | 5 |
| GSM1354773 | 27 | Female | 4 |
